# Lysophosphatidic Acid Acyltransferase Beta Regulates mTOR Signaling

**DOI:** 10.1371/journal.pone.0078632

**Published:** 2013-10-31

**Authors:** Michelle A. Blaskovich, Vimala Yendluri, Harshani R. Lawrence, Nicholas J. Lawrence, Saïd M. Sebti, Gregory M. Springett

**Affiliations:** 1 Department of Experimental Therapeutics, H. Lee Moffitt Cancer Center and Research Institute, Tampa, Florida, United States of America; 2 Department of Drug Discovery, H. Lee Moffitt Cancer Center and Research Institute, Tampa, Florida, United States of America; 3 Department of Gastrointestinal Oncology, H. Lee Moffitt Cancer Center and Research Institute, Tampa, Florida, United States of America; 4 Department of Chemical Biology Core, H. Lee Moffitt Cancer Center and Research Institute, Tampa, Florida, United States of America; 5 Departments of Oncologic Sciences, University of South Florida, Tampa, Florida, United States of America; 6 Molecular Medicine, University of South Florida, Tampa, Florida, United States of America; H.Lee Moffitt Cancer Center & Research Institute, United States of America

## Abstract

Lysophosphatidic acid acyltransferase (LPAAT-β) is a phosphatidic acid (PA) generating enzyme that plays an essential role in triglyceride synthesis. However, LPAAT-β is now being studied as an important regulator of cell growth and differentiation and as a potential therapeutic target in cancer since PA is necessary for the activity of key proteins such as Raf, PKC-ζ and mTOR. In this report we determine the effect of LPAAT-β silencing with siRNA in pancreatic adenocarcinoma cell lines. We show for the first time that LPAAT-β knockdown inhibits proliferation and anchorage-independent growth of pancreatic cancer cells. This is associated with inhibition of signaling by mTOR as determined by levels of mTORC1- and mTORC2-specific phosphorylation sites on 4E-BP1, S6K and Akt. Since PA regulates the activity of mTOR by modulating its binding to FKBP38, we explored the possibility that LPAAT-β might regulate mTOR by affecting its association with FKBP38. Coimmunoprecipitation studies of FKBP38 with mTOR show increased levels of FKBP38 associated with mTOR when LPAAT-β protein levels are knocked down. Furthermore, depletion of LPAAT-β results in increased Lipin 1 nuclear localization which is associated with increased nuclear eccentricity, a nuclear shape change that is dependent on mTOR, further confirming the ability of LPAAT-β to regulate mTOR function. Our results provide support for the hypothesis that PA generated by LPAAT-β regulates mTOR signaling. We discuss the implications of these findings for using LPAAT-β as a therapeutic target.

## Introduction

Phosphatidic acid (PA), is a diacyl glycerolipid second-messenger that functions as a cofactor in several critical signaling pathways that are relevant to cancer cells. PA binds to a polybasic domain of mTOR and is essential for its full activation [1]. Without PA binding, mTOR cannot play its critical role in signaling through its downstream effectors, S6 Ribosomal Kinase (S6K), 4E-BP1, and AKT, which in turn mediate cell growth, differentiation, and survival. Hence the role of PA is central to the regulation of proteins in both proliferative and survival pathways in tumor cells. Cells can produce PA in several ways: the enzymatic conversion of phosphatidylcholine (PC) to PA and choline by phospholipase D (PLD) [2]; diacylglycerol kinase (DAGK) can phosphorylate diacylglycerol (DAG) to produce PA [3]; lysophosphatidic acid acyltransferase (LPAAT) generates PA from lysophosphatidic acid (LPA) by acylating it at the *sn*-2 position [4]. 

A total of five human LPAAT isoforms have been identified, designated LPAAT-α, β, γ, δ, and ε [4-6]. Of these, only LPAAT-α and LPAAT-β have been studied in detail. Though these two enzymes have 48% amino acid homology and share four highly conserved lysophospholipid acyltransferase (LPLAT) domains essential to their enzymatic activities [7], they appear to have non-redundant roles in human tissue. The two enzymes preferentially catalyze the addition of different species of acyl-CoA to LPA [8]. LPAAT-α mRNA has been shown to be expressed ubiquitously [9], whereas LPAAT-β is more specific, with the highest levels of expression being detected in adipose, liver, heart, and pancreas tissue [8]. In addition, mutation of the LPAAT-β gene, leading to loss of enzymatic function, is recognized as the cause of congenital generalized lipodystrophy 1 (CGL1, or Berardinelli-Seip Syndrome) [10,11], a disorder which manifests itself as an almost complete lack of body fat with severe insulin resistance in affected individuals. 

 Another distinction between these two enzymes is that increased expression of LPAAT-β has been linked to a malignant state, while LPAAT-α, has been shown to be expressed at relatively equal levels in both normal and tumor tissue[4]. Past research has identified the upregulation of LPAAT-β mRNA in tumor cells compared to normal tissue in a variety of tumor types, including lung, breast, colon, prostate, and gliomas [12], and human osteosarcoma cells have been shown to overexpress LPAAT-β mRNA and protein[13]. Additionally, LPAAT-β protein has been shown to be upregulated in ovarian and endometrial cancers [14]. Inhibition of LPAAT-β by either pharmacological means or siRNA has been linked to antitumor activity in prostate cancer, leukemia, lymphoma, multiple myeloma, and ovarian carcinoma cells [12,14-17]. Ectopic overexpression of LPAAT-β has been shown to stimulate proliferation of LNCaP prostate cancer cells [12]. 

Several recent studies have demonstrated a link between the PA produced by PLD and DAG Kinase to signaling pathways involved in cancer progression. Both PLD and DAGK have been implicated in mTOR signaling through their ability to produce PA [18,19]. mTOR is a universally-conserved, major signaling protein in eukaryotes, regulating nutrient sensing and uptake, cell growth, protein synthesis, and autophagy and nuclear shape. mTOR exists in two complexes, mTOR complex 1 (mTORC1), which acts through such substrates as 4E-BP1 and S6K to control DNA transcription and protein translation [20] and mTORC2 which phosphorylates AKT on its Ser473 residue, fully activating that kinase for its role in cell proliferation metabolism, and survival [21]. PA regulates the activation of mTORC1 by binding to the FRB (FKBP12-rapamycin binding) domain. One mechanism by which this occurs is displacement of FKBP38 which is a cellular inhibitor of mTOR[22].

Up until recently, it was assumed that the PA produced by LPAAT-β was used only in triglyceride synthesis in adipocytes. Increasingly, as with PLD- and DAGK-produced PA, it appears that PA produced by LPAAT-β also has a signaling function. Injection of LPAAT-β mRNA into *Xenopus* oocytes is able to cooperate with Ras and Raf to enhance Erk activation in a meiotic maturation assay. Conversely, inhibition of LPAAT-β expression with siRNA in mammalian cells suppresses basal Erk phosphorylation [23]. We and others have shown that highly selective small-molecule inhibitors of LPAAT-β can inhibit proliferation and suppress the activation of proteins in the phosphoinositide-3-kinase (PI3K)/AKT pathway, including AKT, mTOR, and S6K in human microvascular endothelial cells and ovarian cancer cell lines [14]. 

In this report, we now show that inhibition of LPAAT-β protein expression in pancreatic adenocarcinoma cells with siRNA, reduces proliferation and anchorage-independent growth. This is associated with enhanced association of FKBP38 with mTOR, resulting in diminished mTOR signaling via 4E-BP1, S6K, and AKT. Our results add LPAAT-β to the list of PA generating enzymes involved in mTOR signaling and provide further evidence that LPAAT-β is a potential tumor-promoting protein, and validate LPAAT-β as a molecular target for drug discovery in pancreatic cancer.

## Results

### Treatment with siRNA to LPAAT-β results in a concentration- and time-dependent decrease in LPAAT-β protein levels in pancreatic cancer cell lines

**Figure 1:**
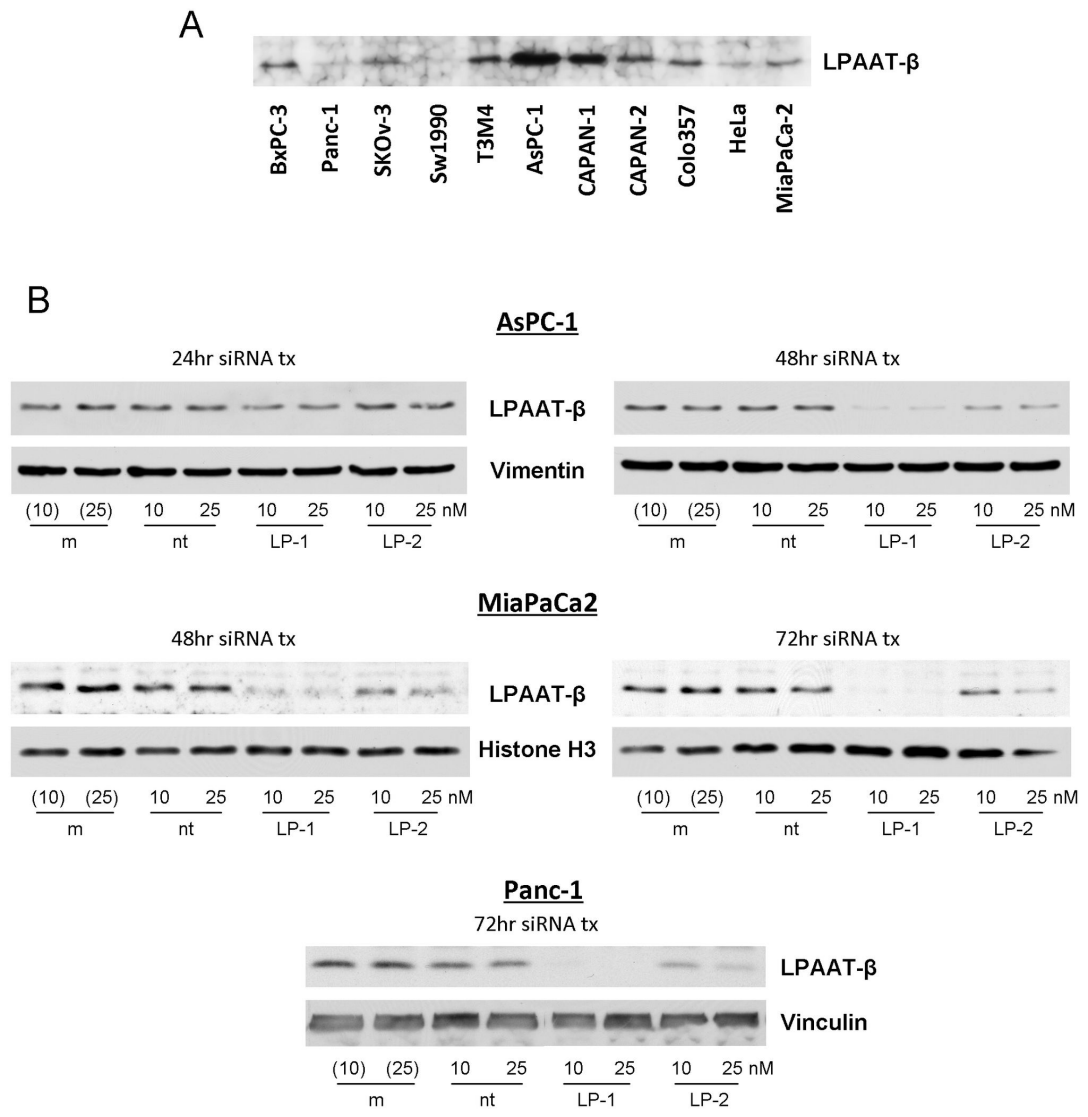
LPAAT-β expression and siRNA knockdown in human pancreatic cancer cells. (A) A panel of human pancreatic cancer cell lines shows differential expression of LPAAT-β protein. (B) Knockdown efficiency of siRNA transfection of AsPC-1, MiaPaCa2, and Panc-1 cells with LPAAT-β siRNA (LP-1 and LP-2) compared to mock transfection (m) and non-targeting (nt) siRNA controls.

LPAAT-β protein was found to be expressed in several pancreatic adenocarcinoma cell lines similar to its previously reported expression in gynecologic cancer cells (Figure 1A) [14]. For our work, we chose three pancreatic cancer cell lines, with high (AsPC-1), intermediate (MiaPaCa2), and low (Panc-1) levels of LPAAT-β protein expression. We used three siRNA constructs targeting LPAAT-β to knock down the expression of LPAAT-β protein, LP-1, LP-2 and LP-4. All three siRNAs exhibited concentration- and time-dependent inhibition. LP-1 and LP-4 (25 nM) inhibited the expression of LPAAT-β by greater than 80% after 72 hours (Figure 1B, Figure S1). LP-2 (25 nM) inhibited expression by greater than 60% after 72 hours of treatment (Figure 1B). 

### Knockdown of LPAAT-β protein expression inhibits anchorage-dependent proliferation of pancreatic cancer cells

**Figure 2:**
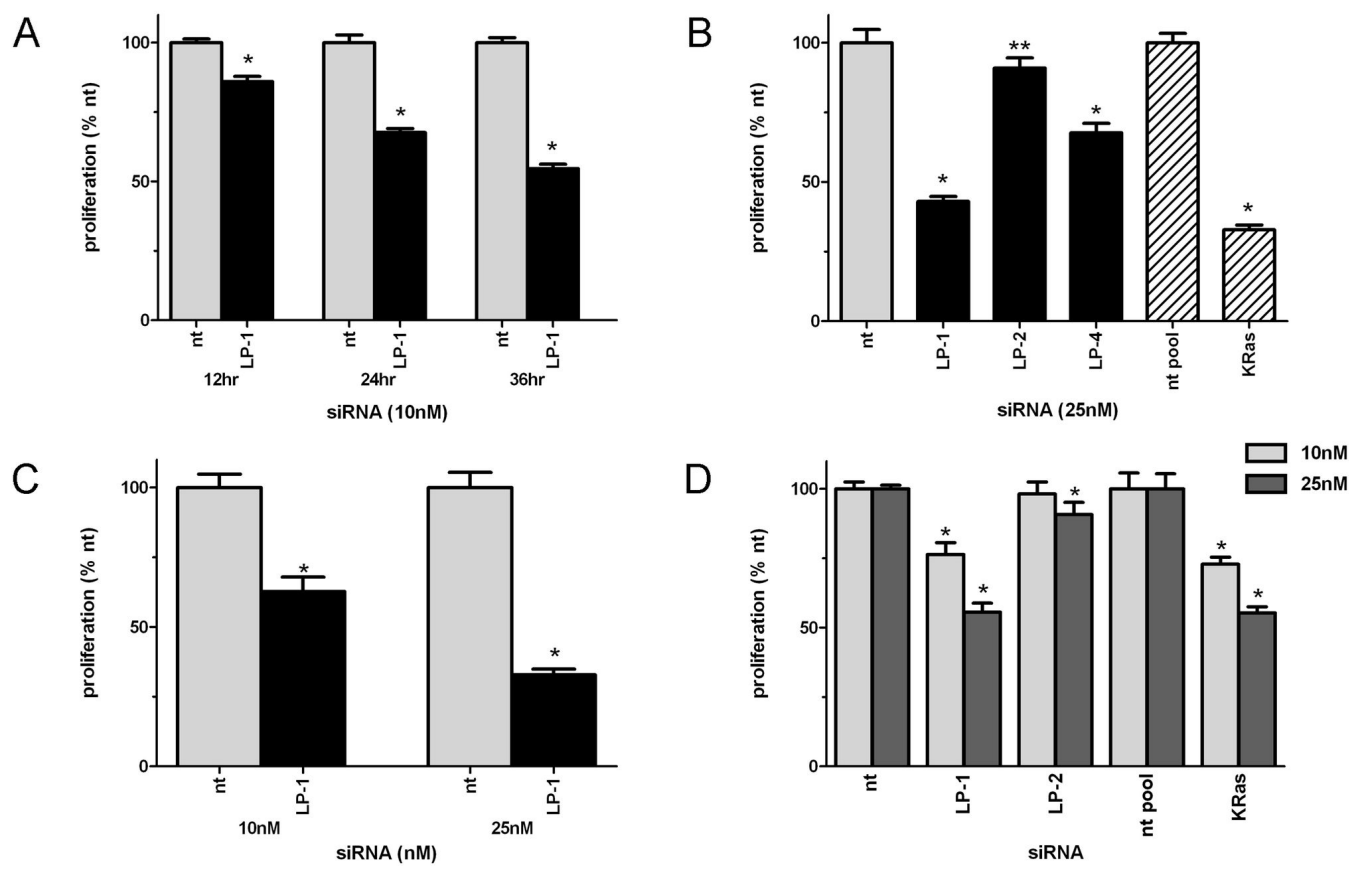
Knockdown of LPAAT-β protein expression results in inhibition of anchorage-dependent proliferation of pancreatic cancer cells. Inhibition of proliferation in (A) AsPC-1 cells transfected with 10 nM siRNA to LPAAT-β (LP-1) and non-targeting (nt) control; (B) AsPC-1 cells transfected for 72 hr with 25 nM non-targeting siRNA, LPAAT-β siRNA (LP-1, LP-2, LP-4), or pooled KRas siRNA; (C) Panc-1 cells transfected for 72 hr with 10 nM and 25 nM non-targeting or LPAAT-β siRNA; (D) MiaPaCa2 cells transfected for 72 hr with 25 nM non-targeting, LPAAT-β, or pooled KRas siRNA. Experiments are representative of at least three replicates. Significance was determined using Student’s t-test, * p < 0.0001; ** p < 0.01; non-targeting (nt, nt-pool); LPAAT-β (LP-1, LP-2, LP-4).

Treatment of pancreatic cancer cell lines with siRNA to LPAAT-β resulted in a time- (Figure 2A) and concentration-dependent (Figure 2C, 2D) inhibition of proliferation that correlated with the degree of protein inhibition. LP-1 at 25 nM for 72 hours resulted in statistically significant inhibition (p < 0.0001) of proliferation of 55% in AsPC-1 (Figure 2B), 70% in Panc-1 (Figure 2C) and 45% in MiaPaCa2 (Figure 2D). Since proliferation in pancreatic cancer is driven primarily by KRas[24], we used siRNA to KRas as a positive control. The degree of inhibition seen with LPAAT-β siRNA was comparable to that seen with treatment with an siRNA to KRas under the same conditions, 60% in AsPC-1 (Figure 2B) and 50% MiaPaCa2 (Figure 2D). The inhibition of proliferation was less, yet still statistically significant with LP-2 and LP-4, as these siRNAs are less effective in knocking down LPAAT-β expression.

### Knockdown of LPAAT-β protein expression results in inhibition of anchorage-independent growth of pancreatic cancer cells

**Figure 3:**
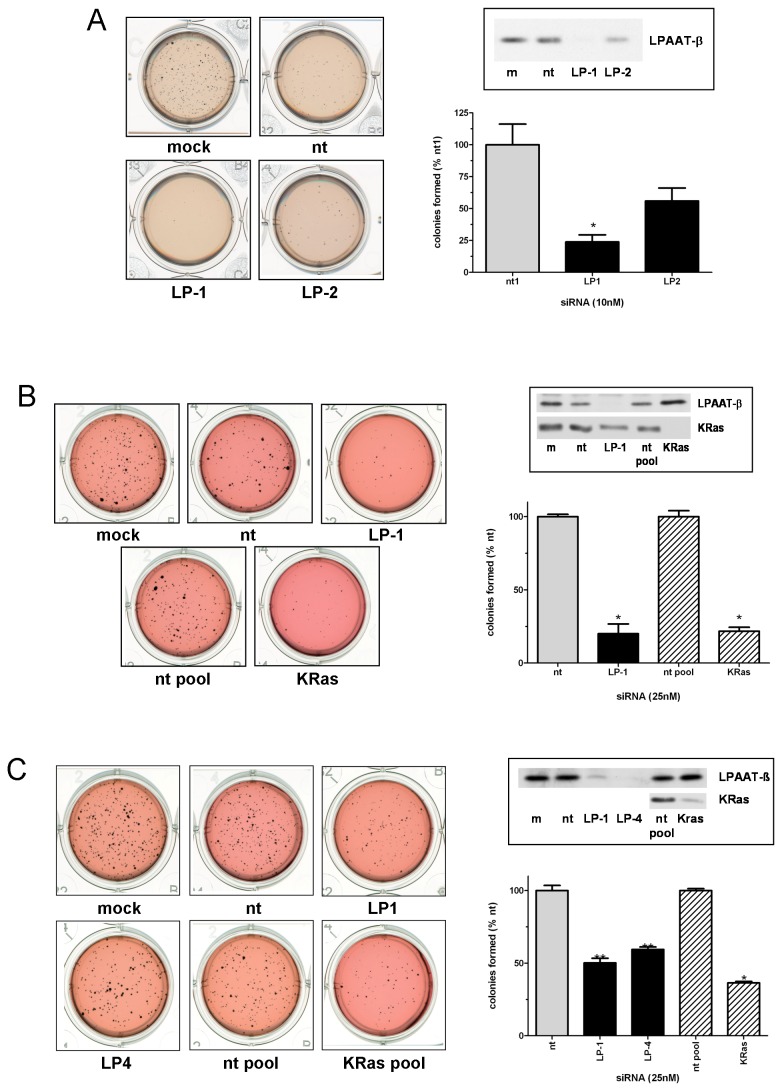
Knockdown of LPAAT-β protein expression results in inhibition of anchorage-independent growth of pancreatic cancer cells. Inhibition of anchorage-independent growth was determined by plating (A) AsPC-1, (B) Panc-1, or (C) MiaPaCa2 cells onto a soft agar matrix after 48 hr (AsPC-1) or 72 hr (Panc-1 and MiaPaCa2) transfection with the indicated siRNA (non-targeting: nt and nt pool; LPAAT-β: LP-1, LP-2, LP-4; and KRas. Insets show confirmation of the knockdown of target proteins. Statistical significance was measured using Student’s t-test comparing the target siRNA samples to their appropriate nt control siRNA; * p < 0.0001; ** p = 0.0005.

To establish whether LPAAT-β also contributes to the ability of cells to grow in an anchorage-independent manner, we transfected pancreatic cancer cells for 72 hours with siRNA to LPAAT-β, harvested them, then plated them for soft agar colony formation assays, allowing 2-4 weeks for the colonies to form and grow. Figure 3A shows the effects of LPAAT-β siRNA on AsPC-1 cells, with both LP-1 and LP-2 inhibiting the formation of colonies in soft agar (p < 0.05 for LP-1). To determine the effect of LPAAT-β siRNA on anchorage-independent growth relative to a known contributor to tumorigenesis in pancreatic cancer, we next transfected Panc-1 cells with both LPAAT-β siRNA and KRas siRNA for 72 hours and plated soft agar assays as with AsPC-1. After two weeks of growth, LPAAT-β siRNA (LP-1) shows an equal efficacy to KRas siRNA at inhibiting the formation of Panc-1 colonies in agar (p < 0.05). As well, 72 hours transfection of MiaPaCa2 cells (Figure 3C) with two different LPAAT-β siRNAs inhibits two week soft agar colony formation to a similar degree as does KRas siRNA (p < 0.05). This data, along with the anti-proliferative effect of silencing LPAAT-β protein translation, point to a possible tumor promoting role for LPAAT-β in pancreatic cancer cells.

### Inhibition of LPAAT-β results in inhibition of phosphorylation of mTOR substrates 4E-BP1, S6K, and AKT

Having demonstrated that loss of LPAAT-β expression inhibits both anchorage-dependent proliferation and anchorage-independent cell growth, we next investigated the mechanism by which the silencing of LPAAT-β affects cellular growth signals. Among the proteins known to require phosphatidic acid for their full activation, mTOR is a well-established contributor to both anchorage-dependent and -independent cell growth [25,26] through its effectors S6K, 4E-BP1, and AKT. mTOR kinase activity phosphorylates (among other sites) Ser65 of 4E-BP1, Thr389 of S6K, and Ser473 of AKT [21,27]. Since PA produced by PLD has been shown to be required for mTOR kinase activity [1,28], we determined whether the PA produced by LPAAT-β might also be required for mTOR effector phosphorylation by depleting LPAAT-β from the cells by siRNA transfection. 

**Figure 4:**
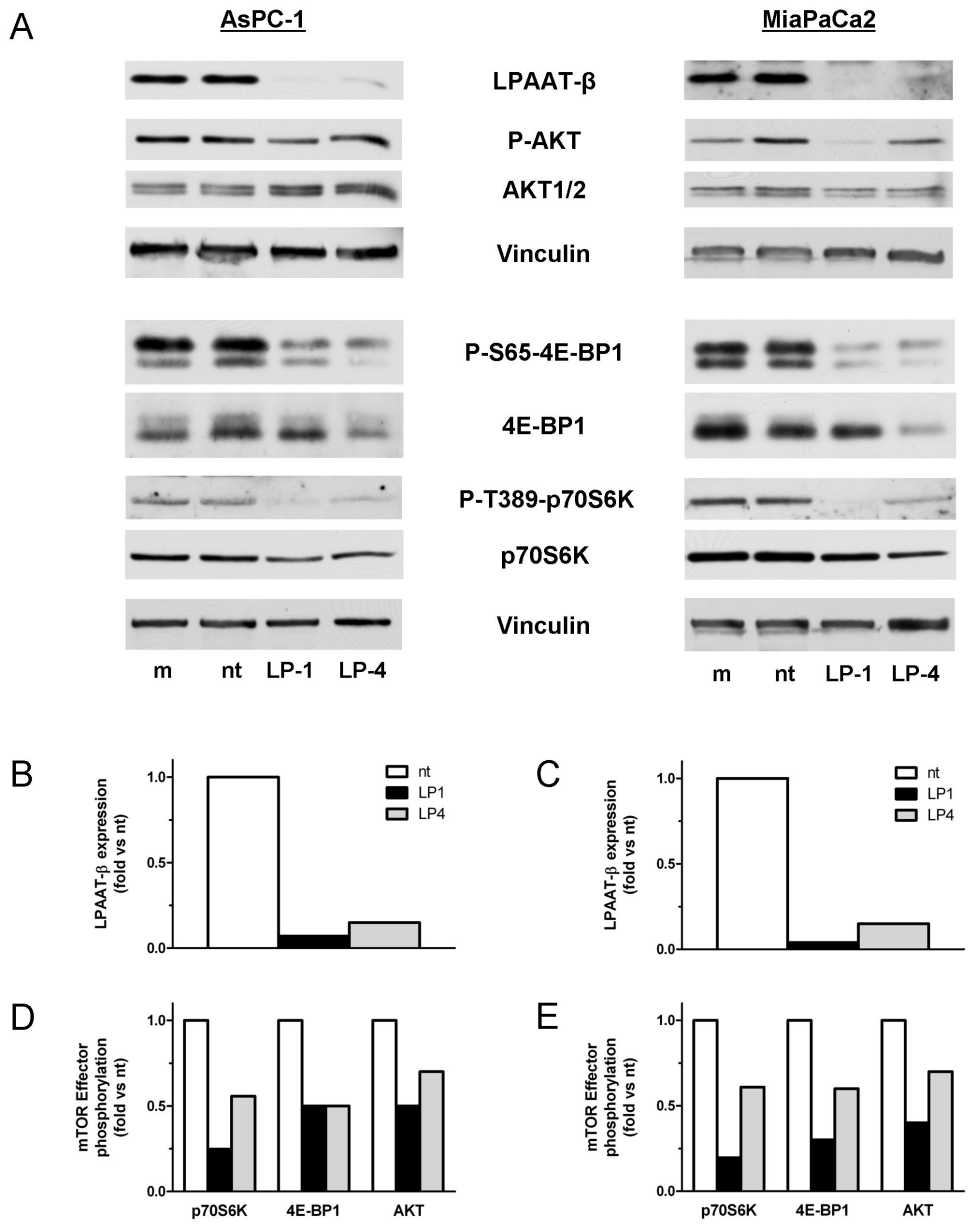
Treatment with LPAAT-β siRNA results in inhibition of phosphorylation of mTOR effector proteins 4E-BP1, S6K, and AKT. AsPC-1 (A) and MiaPaCa2 (B) cells were treated for 72 hr with 25 nM LP-1, LP-4, non-targeting siRNA (nt), or a mock transfectant (m). Western blots were done with the indicated antibodies to mTOR effector proteins and mTOR-specific phosphorylation sites, as well as a Vinculin loading control. The graphs show densitometric quantitation of the phosphorylated bands normalized to both Vinculin and their corresponding whole protein. Results are representative of three independent experiments.

We transfected AsPC-1 and MiaPaCa2 cells with LPAAT-β or non-targeting siRNA and then probed for the mTOR kinase phosphorylation sites of S6K, 4E-BP1, and AKT by Western blotting (Figure 4A). LP-1 and LP-4 inhibited Ser65 phosphorylation of 4E-BP1 by 80% and 50%, respectively in AsPC-1 compared to non-targeting control siRNA (Figure 4A, 4D). In MiaPaCa2 cells the inhibition of phospho-4E-BP1 was 70% and 40% respectively (Figure 4A, 4E). The other mTORC1 kinase target, S6K, was also inhibited at Thr389 by 75% and 50% (Figure 4A, 4D) in AsPC-1 and 80% and 40% in MiaPaCa2 (Figure 4A, 4E). Phosphorylation of the mTORC2 kinase substrate, AKT was inhibited at Ser473 by 50% and 30% respectively in AsPC-1 (Figure 4A, 4B), while in MiaPaCa2 the degree of inhibition was 60% and 40%, respectively (Figure 4A, 4E). The degree of inhibition of signaling correlated well with the extent of knockdown LPAAT- β expression in both cell lines (Figure 4B, 4C). These results suggest that LPAAT-β directly regulates the major mTOR signaling targets in pancreatic cancer cells.

### LPAAT-β regulates the association of FKBP38 with mTOR

**Figure 5:**
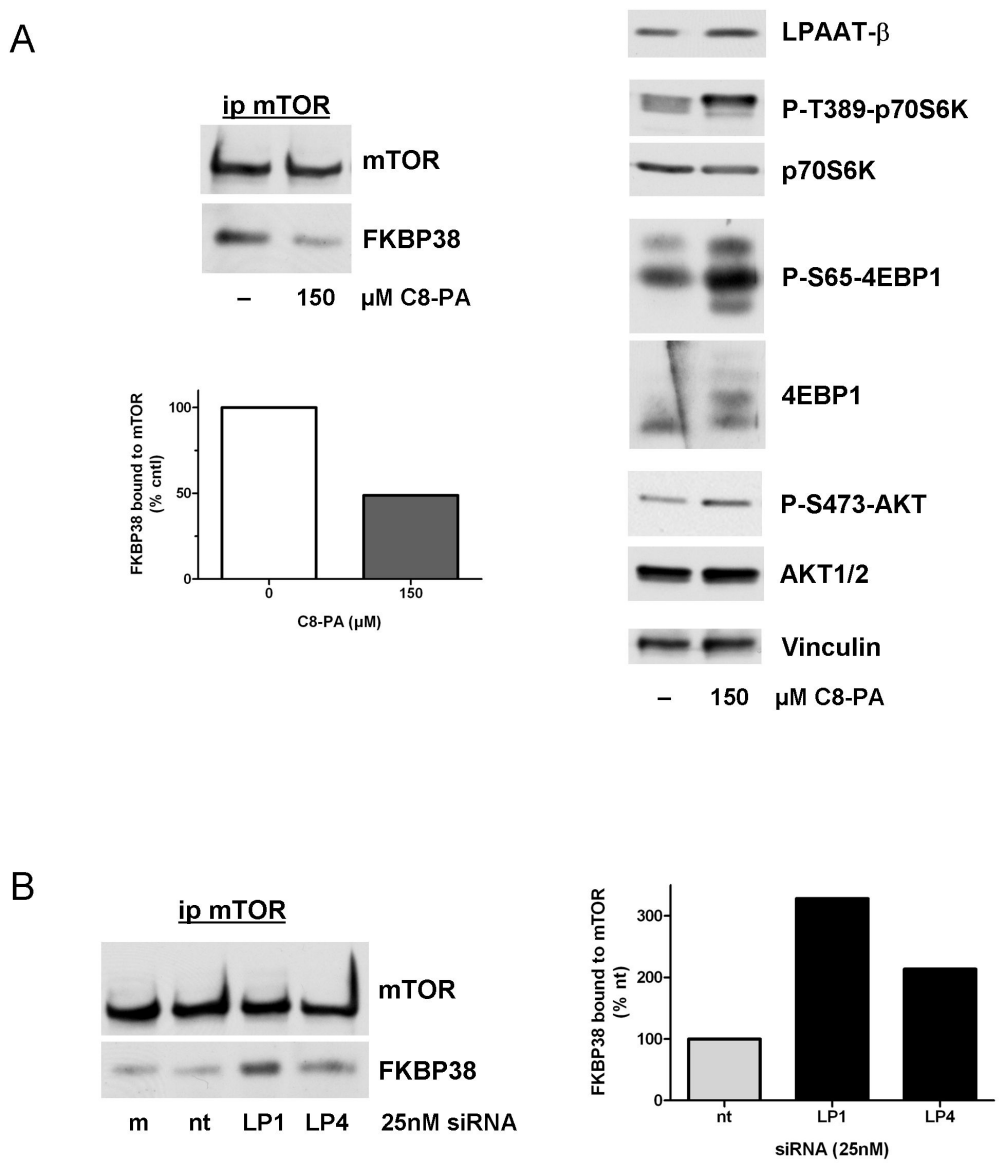
Knockdown of LPAAT-β results in enhancement of FKBP38 with mTOR. (A) 30 min C8-PA treatment of serum-starved AsPC-1 cells results in decreased coimmunoprecipitation of FKBP38 with mTOR and shows enhancement of mTOR signaling via mTOR-specific phosphorylation sites on S6K, 4E-BP1, and AKT. (B) AsPC-1 cells treated for 72 hr with 25 nM LP-1, LP-4, non-targeting (nt), or mock transfection (m) showing enhanced FKBP38 association with mTOR. Results are representative of four independent experiments.

PA and the cellular inhibitor of mTOR, FKBP38, are known to share the same binding site on mTOR, the FRB domain [1]. The mechanism by which PA produced by PLD regulates mTOR is, in part, by displacing FKBP38 from its binding site [22]. Therefore we next examined if LPAAT-β could also affect the ability of FKBP38 to associate with mTOR. First, we confirmed the association of endogenous FKBP38 and mTOR in AsPC-1 cells by co-immunoprecipitation. Treatment of serum-starved cells with cell permeable 1,2-dioctanoyl phosphatidic acid (C8-PA), disrupted the interaction between these molecules by more than 50% in our system (Figure 5A). Published studies have demonstrated this association using recombinant proteins overexpressed after transfection in HEK 293 human embryonic cells, or the endogenous embryonic cell proteins. To our knowledge, this is the first demonstration of this interaction using endogenous proteins in a cancer cell line. This dissociation of FKBP38 from mTOR in the presence of C8-PA is accompanied by an increase in the phosphorylation of S6K, 4E-BP1 and AKT at the sites directly phosphorylated by mTOR (Figure 5B; Figure S2).

To determine the effect of LPAAT-β knockdown on this association, we treated AsPC-1 cells with LPAAT-β siRNA and looked for mTOR-associated FKBP38. We reasoned that if the PA produced by LPAAT-β is utilized by mTOR, then depletion of LPAAT-β should result in decreased PA levels and an increase in the amount of FKBP38 associated with mTOR. Indeed, siRNA to LPAAT-β does reduce cellular levels of PA (Figure S3). Figure 5B shows that, in AsPC-1 cells, transfection with LP-1 and LP-4 siRNAs increased the amount of FKBP38 associated with mTOR by 3- and 2-fold, respectively. This increased association of the inhibitor FKBP38 with mTOR is accompanied by the inhibition of mTOR related signaling shown in Figure 4.

### LPAAT-β regulates nuclear shape and Lipin 1 nuclear localization

To further confirm that LPAAT-β regulates mTOR signaling, we sought out a functional cellular readout that was dependent on both mTOR kinase activity and PA binding. The eccentricity of the nuclear membrane is regulated by Lipin 1 which has a PA binding domain. When bound to PA, Lipin 1 is retained in the cytoplasm [29]. Without phosphorylation of Lipin 1 by mTORC1, Lipin 1 relocalizes to the nucleus where it integrates into the nuclear membrane resulting in elongation of nuclear shape [30]. Pharmacologic manipulations that lower cellular PA level or treatment with Torin-1, an ATP-competitive mTORC1 selective kinase inhibitor can promote this nuclear localization of Lipin 1 and its associated nuclear membrane eccentricity. We reasoned that if LPAAT-β supplies PA to Lipin 1 and mTOR, then LPAAT- β should regulate both nuclear eccentricity and localization of Lipin 1.

**Figure 6:**
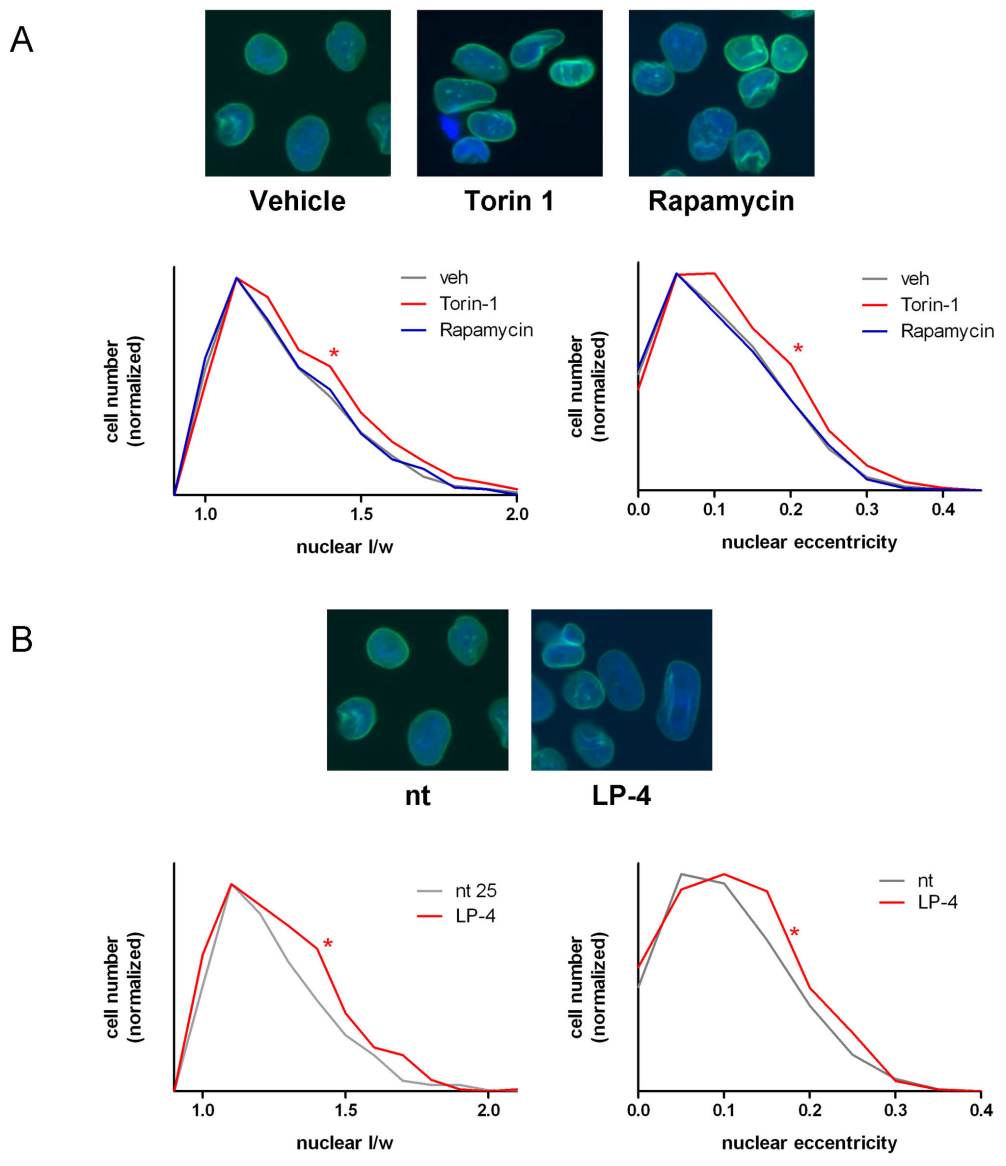
LPAAT-β siRNA treatment results in increased nuclear eccentricity. MiaPaCa2 cells were treated (A) for 24 hr with 500 nM Torin-1 (* p < 0.0001), 10 nM rapamycin, or DMSO vehicle and (B) for 72 hr with 25 nM LP-4 (* p = 0.05) or non-targeting (nt) siRNA. Nuclei were stained with antibody to Lamin A to highlight the nuclear membrane as well as counterstained with DAPI. Analysis of the images using Definiens Tissue Studio software generated the length and width of individual nuclei, which were then analyzed for enhanced nuclear eccentricity as described in Materials and Methods. Graphs show the frequency distribution of nuclear eccentricity and nuclear l/w normalized to cell number. Statistical significance was determined using Student’s t-test. Results are representative of four independent experiments.

To test this, we first established that nuclear eccentricity in MiaPaCa2 cells was increased upon Torin-1 treatment but not rapamycin, as shown by Peterson et al. in NIH 3T3 cells overexpressing Lipin 1[30]. Cells plated onto coverslips were treated for 24 hours either with vehicle control, Torin-1, or rapamycin. Figure 6A shows that MiaPaCa2 cells treated with Torin-1 show the characteristic increase in nuclear eccentricity described previously. To quantify this morphological change, we used two measures of nuclear shape: the formula for eccentricity utilized by Sabatini and colleagues [30], eccentricity = 1-2/((a/p)+1) [30] (see Materials and Methods for an explanation of this formula); and the simple ratio of nuclear length/width. Treatment of MiaPaCa2 cells with Torin-1 shows a measureable, statistically significant increase in nuclear eccentricity as calculated by both measures (p < 0.05 as determined by Student’s t-test). 

**Figure 7:**
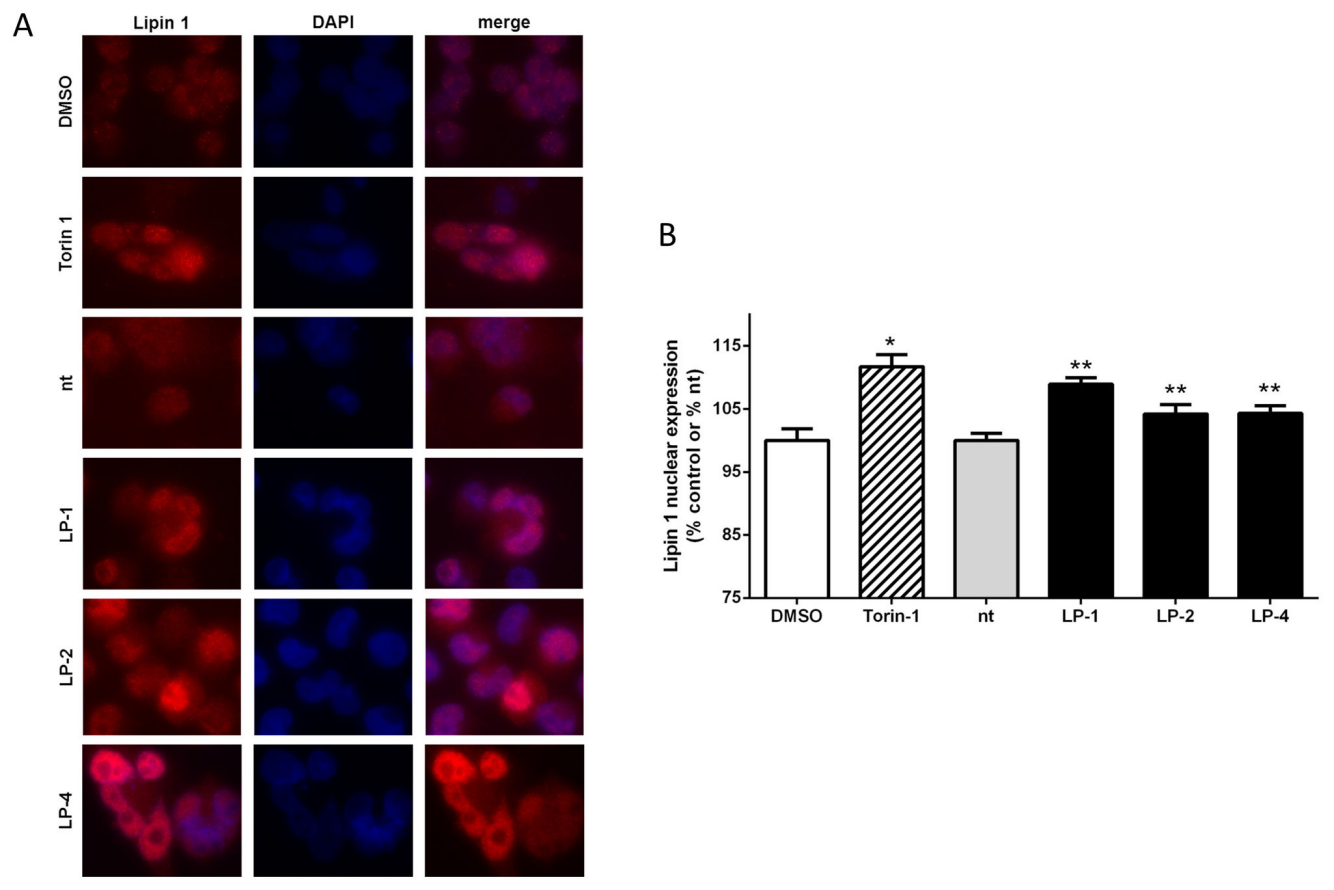
LPAAT-β siRNA treatment results in enhanced nuclear localization of Lipin 1. Immunofluoresence staining (A) for Lipin 1 (red) with the nuclei counterstained with DAPI (blue) and overlay (merge) of MiaPaCa2 cells treated with: DMSO and 500 nM Torin-1 for 24 hours; control siRNA (nt), 10 nM LP-1, 25 nM LP-2 and 10 nM LP-4 for 48 hr. B) Quantitation of nuclear Lipin 1 fluoresence intensity from (A). Statistical significance was calculated using Student’s t-test: DMSO (*) vs Torin-1, p < 0.0001; siRNA nt (**) vs LP-1, p = 0.0005; vs LP-2, p = 0.05; vs. LP-4, p = 0.02. Results are representative of three independent experiments.

 We then transfected MiaPaCa2 cells for 72 hours with either non-targeting siRNA or with siRNA to LPAAT-β (LP-1 and LP-4). Figure 6B reveals that MiaPaCa2 cells treated with LP-4 showed statistically significant nuclear elongation (p < 0.0001), as determined by both measurements used. Likewise, MiaPaCa2 cells transfected for 48 hours with LP-1 or 72 hours with LP-2 also showed statistically significant increases in these measures of nuclear eccentricity (Figure S4). We next assessed the nuclear localization of Lipin 1. Immunostaining of MiaPaCa2 cells treated for 24 hours with Torin-1 show an enhanced localization of Lipin 1 in the nuclei of the cells compared to a diffuse staining of Lipin 1 in DMSO treated cells (Figure 7). Similarly, cells treated for 48 hours with siRNA to LPAAT-β also have enhanced nuclear Lipin 1 staining (Figure 7) that is statistically significant and comparable to the effect of Torin-1. Taken together, these data provide further support for the hypothesis that PA from LPAAT-β regulates the mTOR signaling nexus.

## Discussion

Cellular phospholipids such as phosphatidic acid were originally believed to have roles only in lipid metabolism and membrane biosynthesis. Expanded research in the last decade has elucidated a role for phospholipids in cellular signaling as well. PA is a necessary co-factor for the activity of diverse proteins, such as Raf family proteins, PKC-ζ, RAB11FIP1, PLC-β, SOS and mTOR, which have been implicated in cancer cell proliferation, survival, and tumorigenesis [12,31-34]. Phosphatidic acid produced through PLD enzymatic activity has been shown to contribute to mitogenic signaling cascades such as EGF and insulin, and PA produced by PLD is a regulator of the signaling of small GTPases such as Arf and Rho [35-37]. Likewise, PA produced by DAGK has been shown to regulate the activity of phosphatidylinositol-4-phosphate 5-kinase type Iα (PI5K), and of Toll-like receptor (TLR) signaling in macrophages [3]. The present work now implicates the third cellular producer of PA, LPAAT-β, in a regulatory role of mTOR, a pivotal signaling node that regulates cell growth and survival. The relative contribution of each of these PA generating systems to growth and survival signaling remains to be determined. Interestingly, Ren et al treated human embryonic kidney cells (HEK-293) with two PLD inhibitors (FIPI, CAY10593) and measured cellular PA levels by HPLC tandem mass spectrometry. Decreases of only 20-30% of cellular PA were seen. This implies that the remaining could be supplied by LPAAT-β, other members of the LPAAT protein family, or DAGK [29]. 

To our knowledge, this is the first demonstration that knocking down LPAAT-β using siRNA results in a loss of cell proliferative ability and of the ability of cells to grow in an anchorage-independent manner. It is striking that in both of these assays, knockdown of LPAAT-β was as effective in inhibiting growth as was knockdown of KRas. This finding is of potential translational significance for KRas driven malignancies such as pancreatic ductal adenocarcinoma. There are four dominant effector pathways downstream of KRas: Raf/Mek/Erk, Akt/mTOR, RalGDS/RalA/B, and PLCε/PKC-ζ. The fact that three of these four effector pathways have key proteins (namely, Raf, mTOR, PKC- ζ) that are PA-dependent may suggest how LPAAT-β knockdown could be this effective at inhibiting tumor cell growth. Since therapeutic targeting of KRas has been hampered by the need to inhibit multiple effector pathways simultaneously, targeting of LPAAT-β production of PA may be a strategy to accomplish this using a single inhibitor. 

We have also shown for the first time that LPAAT-β regulates nuclear eccentricity, an important cellular process that is, in part, regulated by Lipin 1, an mTOR substrate. This finding implies that LPAAT-β regulates Lipin 1 localization through PA binding and phosphorylation by mTOR. In this regard, it is interesting to note that loss of function mutations in LPAAT-β and Lipin 1 both result in lipodystrophy and insulin resistance. Loss of either of these proteins impairs adipocyte differentiation. Since Lipin 1 is a phosphatidic acid phosphatase, it may also have a role in terminating the PA signal generated by LPAAT-β. We propose that LPAAT-β be further investigated as a potential target in cancer and obesity therapeutics. 

## Materials and Methods

### Cell lines

AsPC-1, MiaPaCa2, and Panc-1 cell lines were obtained from American Type Culture Collection (Manassas, VA). AsPC-1 cells were maintained in RPMI 1640 medium; MiaPaCa2 and Panc-1 were maintained in Dulbecco’s Modified Eagle Medium.

### siRNA transfection and western blotting

LPAAT-β siRNA coding an internal region of the LPAAT-β mRNA (LP-1, GUGGUGUACUCUUCCUUCUdTdT) or the 5’ untranslated region of LPAAT-β mRNA (LP-2, UCCCGGCUUCCAAAUACCAdTdT) and were synthesized by Dharmacon (Thermo Fisher Scientific, Waltham, MA). A third LPAAT-β siRNA (LP-4, siRNA ID# s223130) and a non-targeting siRNA control (Silencer® Negative Control 1) were acquired from Life Technologies (Carlsbad, CA). Pooled KRas siRNA (siGENOME SMARTpool, Human KRas) and pooled non-targeting siRNA control (siGENOME nontargeting siRNA pool 2) were both acquired from Dharmacon. The cell lines were treated with siRNA as follows: semi-confluent cells were transfected with siRNA at the indicated concentrations with Lipofectamine RNAiMAX (Life Technologies) according to manufacturer’s instructions, using 1µl of transfection reagent per 10 nM siRNA in normal growth medium for the amount of time indicated. Cells were then harvested in cold lysis buffer containing 40mM Hepes, pH 7.2, 120mM sodium chloride, 10mM pyrophosphate, 50mM sodium fluoride, 10mM β-glycerophosphate, 2mM EDTA, and 0.3% CHAPS, with peptidase inhibitors (Complete Mini, Roche Diagnostics, Indianapolis, IN). Equal protein amounts of cleared lysates were run on SDS-PAGE gels, transferred to polyvinylidene difluoride (PVDF) membranes, blocked in 5% non-fat dry milk in Tris-buffered saline, pH 7.4, containing 0.1% Tween-20 (TBS-T), and blotted with the antibodies which follow.

### Antibodies

LPAAT-β antibody was designed to recognize a non-transmembrane sequence of 16 residues corresponding to amino acids 77-92 of both the A and B isoforms of human LPAAT-β (New England Peptide, Gardner, MA). Other antibodies used are all commercially available: phospho-S65-4E-BP1 and mTOR antibodies (Cell Signaling Technology, Danvers, MA); phospho-S473-AKT (Life Technologies); phosphor-T389-p70S6 Kinase and RalA (Epitomics, Burlingame, CA); 4E-BP1, AKT1/2, FKBP38, p70S6 Kinase, and Vimentin (Santa Cruz Biotechnology, Santa Cruz, CA); Histone H3 (Abcam, Cambridge, MA); Vinculin (Sigma-Aldrich, St. Louis, MO); KRas (EMD-Millipore, Billerica, MA).

### Immunoprecipitation of mTOR

For co-immunoprecipitation experiments, cells were transfected with siRNA and harvested as above. For stimulation with water soluble phosphatidic acid, C8-PA (1,2-dioctanoyl-*sn*-glycero-3-phosphate, Avanti Polar Lipids, Alabaster, AL), cells were plated as above, but then starved in serum-free medium for 24-48 hours prior to a 30 minute incubation with C8-PA. Stimulated cells were then immediately washed with ice-cold phosphate-buffered saline (PBS) and lysed in cold lysis buffer as above. Equal amounts of lysates were immunoprecipitated against mTOR antibody (1:350 v/v) overnight at 4°C then proteins were pulled down with Protein A agarose beads (Santa Cruz Biotechnology) for an additional 2 hours at 4°C. Beads were washed three times with lysis buffer before running on SDS-PAGE gels. After transfer to PVDF, western blots were performed using the antibodies above.

### Soft agar and proliferation assays

Cells were tested for anchorage-independent growth using a soft agar assay. Cells were treated with siRNA as described above for 72 hours, then the cells were harvested using Accutase (Innovative Cell Technologies, San Diego, CA), counted, and an equal number plated into a 0.3% agar/medium mixture on top of a 0.6% agar feeder layer in triplicate in twelve-well tissue culture plates. The agar plates were incubated for either two weeks (MiaPaCa2 and Panc-1) or four weeks (AsPC-1) at 37°C in a tissue culture incubator. After completing this incubation, colonies were stained overnight using 1mg/ml MTT (3-(4,5-Dimethyl-2-thiazolyl)-2,5-diphenyl-2H-tetrazolium bromide, EMD Millipore, Billerica, MA), and images were captured using a standard photo scanner. Colonies were counted using Image ProPlus software. Statistical significance was calculated by Student’s t-test.

 For anchorage-dependent cell proliferation assays, cells were transfected with siRNA for 12-72 hours as indicated then harvested as with the soft agar experiment. The cells were counted and an equal number for each condition were also plated in at least quadruplicate into the wells of 96-well plates. The cells were then grown for an additional 72 hours to test the effect of the siRNA on subsequent anchorage-dependent growth. The results were obtained using Alamar blue (Life Technologies) according to the manufacturer’s instructions. Fluorometric results of the Alamar blue assay were read using a Biotek Synergy HT plate reader (Biotek, Winooski, VT). Statistical significance was calculated using Student’s t-test.

### Nuclear eccentricity

To determine the effect of LPAAT-β siRNA treatment on nuclear eccentricity, MiaPaCa2 cells plated onto acid-washed, poly-lysine coated cover slips were treated with siRNA as described above for 48-72 hours. Experimental control cells were treated for 24 hours with either a positive control, Torin-1 (R & D Systems, Minneapolis, MN), a negative control, Rapamycin (Cell Signaling Technologies), or DMSO vehicle. All samples were then fixed onto the cover slips with 4% paraformaldehyde (Electron Microscopy Sciences, Hatfield, PA); cells were permeabilized with 0.2% Triton-X-100 and blocked in 0.25% bovine serum albumin in PBS before staining with antibody to Lamin A (1:200, Santa Cruz Biotechnology) overnight at 4°C. To visualize Lamin A, the cover slips were then incubated with an Alexa Fluor 488-conjugated anti-rabbit secondary (1:750, Life Technologies) for 1 hour at room temperature. Cover slips were mounted onto slides using Vectashield mounting medium with DAPI (Vector Labs, Burlingame, CA), and images of the cell nuclei were taken using a Zeiss automated upright fluorescent microscope and AxioVision microscopy software (Carl Zeiss Vision, Inc., San Diego, CA). Images were analyzed for nuclear shape using Definiens Tissue Studio software (Definiens, Inc., Carlsbad, CA). Nuclear eccentricity was calculated using two separate formulae: first the simple ratio of nuclear length/width; second, the formula described by Sabatini and colleagues[30], eccentricity = 1-2/((a/p)+1), where a is defined as the radius at apoapsis, the farthest distance from the edge of an ellipse to its center, and p is the radius at periapsis, the closest distance from the edge of an ellipse to its center. Using these measurements, a perfectly round nucleus thus has an eccentricity value of zero while elongated nuclei show eccentricity values increasing towards 1; for 1/w ratio, a perfectly round nuclei would have equal length and width and thus a value of 1, whereas increasingly eccentric nuclei would show increasing ratios with values >1. Statistical significance for both measures was calculated using Student’s t-test.

### Analysis of cellular PA content

MiaPaCa2 cells were treated for 24 hours with vehicle (DMSO) control or DAG Kinase inhibitor R59949 (10 µM; Enzo Life Sciences, Farmingdale, NY), or for 48 hours with siRNA to LPAAT-β (LP-1, LP-2, LP-4), mTOR, KRas (pooled siRNA), and non-targeting siRNA control. Cells were harvested and total cellular lipids extracted as follows: Cells were scraped from plates and plates washed twice with cold PBS, pH 7.4, to maximize the number of cells collected. Cells were spun at 1500 x g at 4°C for 10 minutes to collect pellets, which were washed once with 1ml of PBS. After a second spin, this wash was removed and replaced with 1ml per sample of fresh, cold PBS, and the samples were sonicated with a Branson Sonifier 450 (Branson Ultrasonics, Danbury, CT), using a microtip with power setting of 4 and a 70% duty cycle for 10 seconds per sample. Each sample was then subjected to lipid extraction by adding 1.5ml methanol, 2.25ml 1M NaCl, and 2.5ml chloroform to each sample. Samples were vortexed and spun as above to separate the phases. At this point, the upper phase was discarded and the lower phase washed twice with 2ml/wash of preequilibrated upper phase (50:50:45 methanol:chloroform:1M NaCl, mixed well and then allowed to settle into two phases, the upper phase of which – PEU – is used for washes). After each PEU wash, the samples were spun as above. Following the second PEU wash, the upper phase of each sample was discarded and the lower phase removed by needle and syringe to a clean, pre-weighed 12 x 75 mm glass tube. This organic lower phase was dried under a steady stream of nitrogen resulting in a lipid pellet at the bottom of each tube. Tubes were reweighed to determine the weight of the lipid extract and frozen at -20°C prior to MS analysis.

For MS analysis, each sample was resuspended in 1ml methanol and sonicated to make a homogenous suspension of the lipids. Samples were placed into autosampler vials and 1µl per sample analyzed using formula guided High Resolution Mass Spectroscopy (HRMS) carried out on an Agilent 6210 LC-MS (ESI-TOF) machine (Agilent Technologies, Santa Clara, CA). The mass analyses were carried out in the ESI-ve (Electrospray Ionization -ve) mode without a column. A readout of each sample in the range of 600 - 780 m/v was provided for analysis.

Peaks corresponding to 27 PA species previously identified as naturally occurring in mammalian cells[38] were measured and normalized versus the weight of the lipid extracts. The percent inhibition of PA was calculated by comparing each siRNA to its proper non-targeting control siRNA. The PA peaks with m/z of < 700 (z = 1) were insubstantial compared to the background noise of the samples; these peaks were not included in the analysis. The 15 PA peaks between 700 and 752 m/z (z = 1) were summed and an inhibition of PA produced calculated compared to non-targeting siRNA or DMSO control (for DAG Kinase inhibitor). Results are representative of three individual experiments.

### Lipin 1 nuclear staining

To determine the effect of LPAAT-β siRNA treatment on the nuclear localization of Lipin 1, MiaPaCa2 cells plated onto cover slips were treated with siRNA as described previously for 48 hours or with Torin-1 or DMSO vehicle for 24 hours. Cells were fixed, permeabilized, and blocked as described above then incubated overnight with Lipin 1 antibody (1:200, Santa Cruz Biotechnology). To visualize Lipin 1, the cover slips were then incubated with an Alexa Fluor 594-conjugated anti-mouse secondary (1:750, Life Technologies) for 1 hour at room temperature. Coverslips were mounted and visualized, and imaged for intensity of nuclear Lipin 1 fluorescence using Definiens Tissue Studio software. Statistical significance was calculated using Student’s t-test.

## Supporting Information

Figure S1 **LP-4 siRNA treatment blocks the expression of LPAAT-β in a time and concentration dependent manner.** As with LP-1 and LP-2 (LPAAT-β siRNAs shown in Figure 1), we transfected AsPC-1, MiaPaCa2, and Panc-1 human pancreatic cancer cell lines for 48 and 72 hr with 10 nM and 25 nM of either non-targeting or LPAAT-β specific siRNA (LP-4). The response to LP-4 is both time and concentration dependent.
(TIF)

Figure S2 **Quantitation of the Effect of C8-PA treatment on signaling.** Serum-starved AsPC-1 cells were stimulated with 150 µM C8-PA for 30 min. Stimulation was halted by removing the medium and washing cells with ice-cold PBS, pH 7.5, then immediately lysing the cells in ice-cold lysis buffer as described in Materials and Methods. To demonstrate the effectiveness of C8-PA at stimulating mTOR effector pathways, we performed Western blots on these cell lysates (Figure 5A). The graphs show densitometric quantitation of the phosphorylated bands normalized to both Vinculin and their corresponding whole protein. Results are representative of three independent experiments.
(TIF)

Figure S3 **LPAAT-β siRNA, but not siRNA to mTOR or KRas, inhibits the production of PA in MiaPaCa2 cells as measured by Mass Spectrometry.** Whole cell lipid extracts were isolated from MiaPaCa2 cells treated with either DMSO vehicle or DAG Kinase inhibitor (R59949) and with non-targeting siRNA or siRNA specific to either LPAAT-β, mTOR, or KRas. Peaks were analyzed using formula guided High Resolution Mass Spectroscopy (HRMS) carried out on an Agilent 6210 LC-MS (ESI-TOF) machine. Twenty-seven PA species were measured and normalized versus the weight of the lipid extracts. PA peaks < 700 m/z (z = 1) were insubstantial compared to the background noise of the samples and were not included in the analysis. The remaining 15 PA peaks between 700-752 m/z (z = 1) were summed and an inhibition of PA produced was calculated by comparing treated samples to either non-targeting siRNA or DMSO control. The data shown in this figure are representative of three individual experiments.
(TIF)

Figure S4 **48 hr treatment with LP-1 and 72 hr with LP-2 results in a statistically significant increase in nuclear eccentricity of MiaPaCa2 cells.** (A) The more potent and faster-acting LP-1 peaked in its effect on nuclear eccentricity at an earlier time point than 72 hr treatment used for Figure 6 of the main text. MiaPaCa2 cells were treated as described in Materials and Methods with 10nM or 25 nM LP-1 and 25 nM LP-2 for 48 hr. Staining of the cells and analysis revealed that LP-1 treatment of MiaPaCa2 cells shows a significant effect on increasing nuclear eccentricity by both measures (l/w ratio and eccentricity formula as described in Materials and Methods) at 25 nM. 10 nM LP-1 treatment also shows the same effect, but does not achieve statistical significance according to Student’s t-test. (B) 72 hr treatment with LP-2 siRNA to LPAAT-β also results in a statistically significant increase in nuclear eccentricity by both measures. (* p = 0.01, Student’s t-test).
(TIF)
