# Supplementary figures and images for: Lysophosphatidic Acid Acyltransferase Beta Regulates mTOR Signaling
Source: PLoS One. 2013 Oct 31;8(10):e78632. doi: 10.1371/journal.pone.0078632 (PMC3814986; doi:10.1371/journal.pone.0078632)

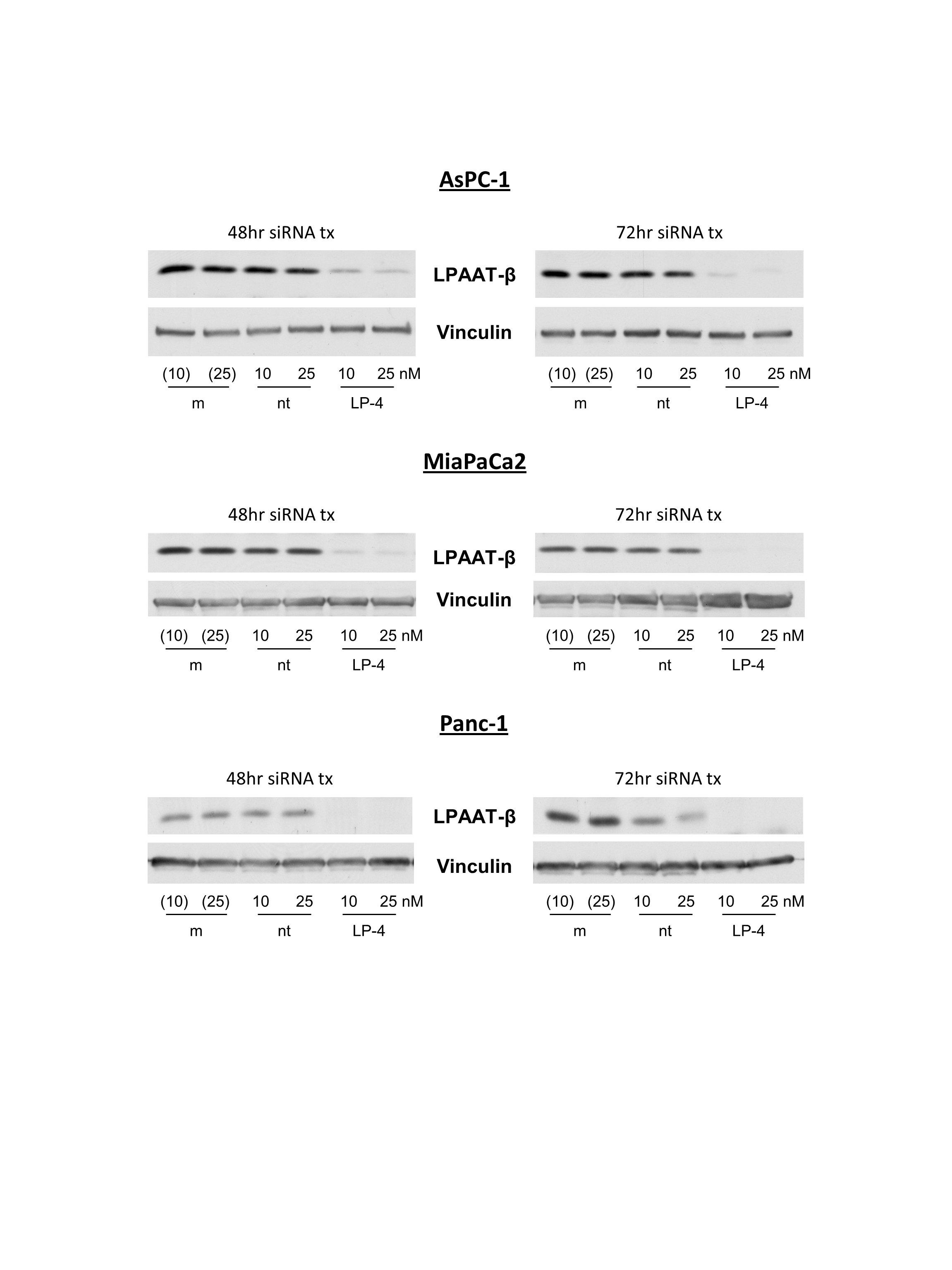

Supplement: Figure S1 — LP-4 siRNA treatment blocks the expression of LPAAT-β in a time and concentration dependent manner. As with LP-1 and LP-2 (LPAAT-β siRNAs shown in Figure 1), we transfected AsPC-1, MiaPaCa2, and Panc-1 human pancreatic cancer cell lines for 48 and 72 hr with 10 nM and 25 nM of either non-targeting or LPAAT-β specific siRNA (LP-4). The response to LP-4 is both time and concentration dependent. (TIF) [file pone.0078632.s001.tif]

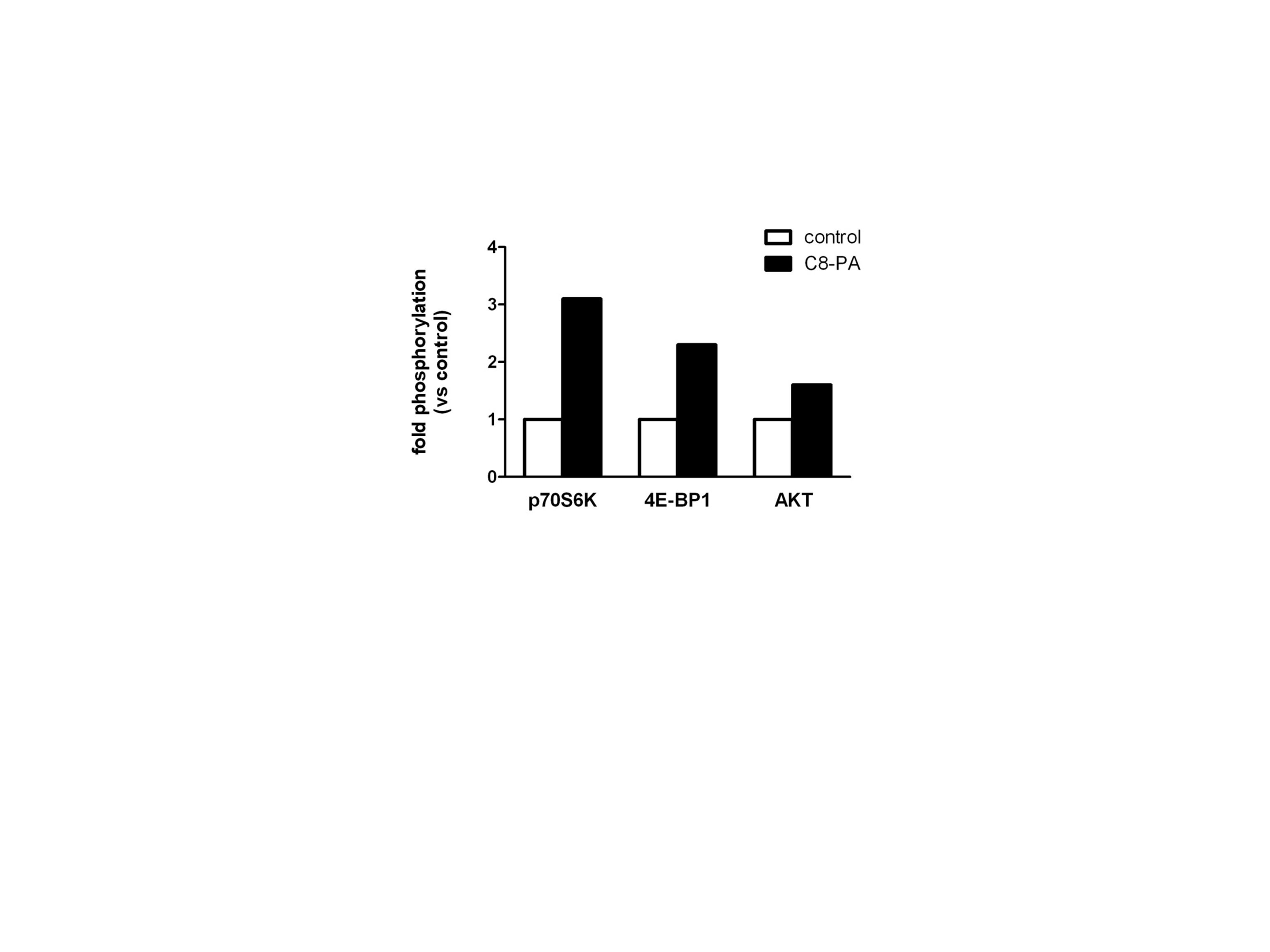

Supplement: Figure S2 — Quantitation of the Effect of C8-PA treatment on signaling. Serum-starved AsPC-1 cells were stimulated with 150 µM C8-PA for 30 min. Stimulation was halted by removing the medium and washing cells with ice-cold PBS, pH 7.5, then immediately lysing the cells in ice-cold lysis buffer as described in Materials and Methods. To demonstrate the effectiveness of C8-PA at stimulating mTOR effector pathways, we performed Western blots on these cell lysates (Figure 5A). The graphs show densitometric quantitation of the phosphorylated bands normalized to both Vinculin and their corresponding whole protein. Results are representative of three independent experiments. (TIF) [file pone.0078632.s002.tif]

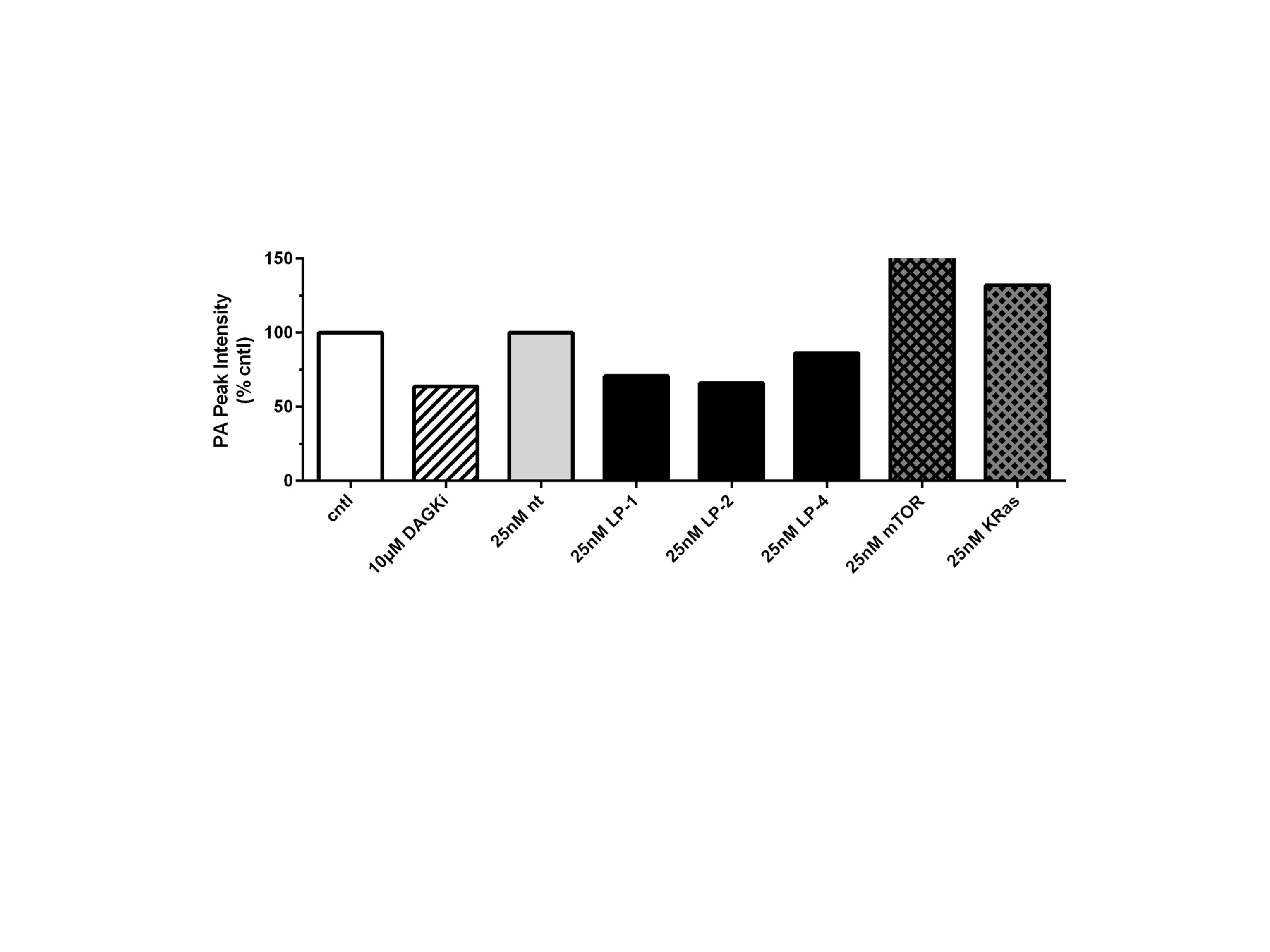

Supplement: Figure S3 — LPAAT-β siRNA, but not siRNA to mTOR or KRas, inhibits the production of PA in MiaPaCa2 cells as measured by Mass Spectrometry. Whole cell lipid extracts were isolated from MiaPaCa2 cells treated with either DMSO vehicle or DAG Kinase inhibitor (R59949) and with non-targeting siRNA or siRNA specific to either LPAAT-β, mTOR, or KRas. Peaks were analyzed using formula guided High Resolution Mass Spectroscopy (HRMS) carried out on an Agilent 6210 LC-MS (ESI-TOF) machine. Twenty-seven PA species were measured and normalized versus the weight of the lipid extracts. PA peaks < 700 m/z (z = 1) were insubstantial compared to the background noise of the samples and were not included in the analysis. The remaining 15 PA peaks between 700-752 m/z (z = 1) were summed and an inhibition of PA produced was calculated by comparing treated samples to either non-targeting siRNA or DMSO control. The data shown in this figure are representative of three individual experiments. (TIF) [file pone.0078632.s003.tif]

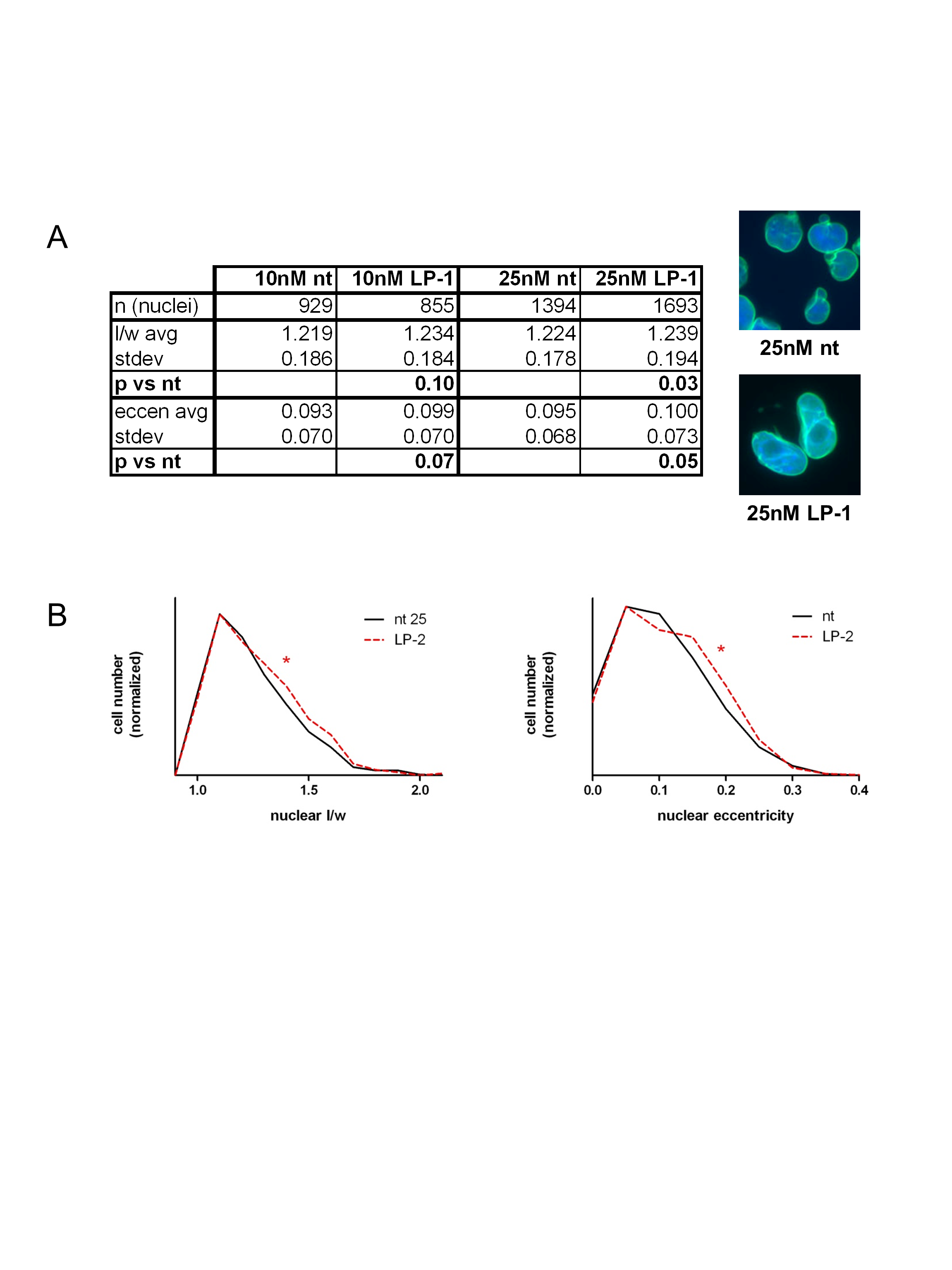

Supplement: Figure S4 — 48 hr treatment with LP-1 and 72 hr with LP-2 results in a statistically significant increase in nuclear eccentricity of MiaPaCa2 cells. (A) The more potent and faster-acting LP-1 peaked in its effect on nuclear eccentricity at an earlier time point than 72 hr treatment used for Figure 6 of the main text. MiaPaCa2 cells were treated as described in Materials and Methods with 10nM or 25 nM LP-1 and 25 nM LP-2 for 48 hr. Staining of the cells and analysis revealed that LP-1 treatment of MiaPaCa2 cells shows a significant effect on increasing nuclear eccentricity by both measures (l/w ratio and eccentricity formula as described in Materials and Methods) at 25 nM. 10 nM LP-1 treatment also shows the same effect, but does not achieve statistical significance according to Student’s t-test. (B) 72 hr treatment with LP-2 siRNA to LPAAT-β also results in a statistically significant increase in nuclear eccentricity by both measures. (* p = 0.01, Student’s t-test). (TIF) [file pone.0078632.s004.tif]
